# Fluorescent protein tagging of endogenous protein in brain neurons using CRISPR/Cas9-mediated knock-in and *in utero* electroporation techniques

**DOI:** 10.1038/srep35861

**Published:** 2016-10-26

**Authors:** Takeshi Uemura, Takuma Mori, Taiga Kurihara, Shiori Kawase, Rie Koike, Michiru Satoga, Xueshan Cao, Xue Li, Toru Yanagawa, Takayuki Sakurai, Takayuki Shindo, Katsuhiko Tabuchi

**Affiliations:** 1 Department of Molecular and Cellular Physiology, Institute of Medicine, Academic Assembly, Shinshu University, Nagano 390-8621, Japan; 2 Institute for Biomedical Sciences, Interdisciplinary Cluster for Cutting Edge Research, Shinshu University, Nagano 390-8621, Japan; 3 CREST, JST, Saitama 332-0012, Japan; 4 Department of Oral and Maxillofacial Surgery, Faculty of Medicine, University of Tsukuba, Ibaraki 305-8575, Japan; 5 Department of Cardiovascular Research, Shinshu University Graduate School of Medicine, Nagano 390-8621, Japan; 6 PRESTO, JST, Saitama 332-0012, Japan

## Abstract

Genome editing is a powerful technique for studying gene functions. CRISPR/Cas9-mediated gene knock-in has recently been applied to various cells and organisms. Here, we successfully knocked in an EGFP coding sequence at the site immediately after the first ATG codon of the β-actin gene in neurons in the brain by the combined use of the CRISPR/Cas9 system and *in utero* electroporation technique, resulting in the expression of the EGFP-tagged β-actin protein in cortical layer 2/3 pyramidal neurons. We detected EGFP fluorescence signals in the soma and neurites of EGFP knock-in neurons. These signals were particularly abundant in the head of dendritic spines, corresponding to the localization of the endogenous β-actin protein. EGFP knock-in neurons showed no detectable changes in spine density and basic electrophysiological properties. In contrast, exogenously overexpressed EGFP-β-actin showed increased spine density and EPSC frequency, and changed resting membrane potential. Thus, our technique provides a potential tool to elucidate the localization of various endogenous proteins in neurons by epitope tagging without altering neuronal and synaptic functions. This technique can be also useful for introducing a specific mutation into genes to study the function of proteins and genomic elements in brain neurons.

Information of the precise localization of proteins is essential for elucidating their functions, because protein localization and function are closely related. Immunohistochemical analysis using specific antibodies has been widely used to examine protein localization, but it is often accompanied by difficulties such as the specificity and sensitivity of antibodies. Alternatively, transient overexpression of an epitope-tagged protein in cells has been used to investigate the subcellular distribution of proteins. However, overexpression of proteins often causes artificial mislocalizations of expressed proteins or perturbs normal cellular functions^1^,^2^. The knock-in of an epitope tag at genomic loci by homologous recombination is one of the methods used to overcome such problems and has been applied to cultured cells^3^, but its extremely low homologous recombination efficiency is an obstacle to the *in vivo* of application of this method.

Conventional homologous recombination has been used to introduce an epitope tag into a genomic locus in embryonic stem (ES) cells^4^. Genetic modification of mice using recombinant ES cells enables the expression of epitope-tagged endogenous proteins and analysis of the localization of endogenous proteins *in vivo*. However, establishment of genetically modified mice from recombinant ES cells is often costly and time-consuming. The recent discovery of the type II bacterial clustered regularly interspaced short palindromic repeat (CRISPR) and CRISPR-associated protein 9 (CRISPR/Cas9) system enables simple and highly efficient genome editing in a wide variety of cells and organisms of different species^5^,^6^. Cas9 combined with single-guide RNA (sgRNA) recognizes a target genomic sequence proximal to a protospacer adjacent motif (PAM) and induces site-specific DNA double-strand breaks (DSBs), which can be repaired by nonhomologous end joining (NHEJ) or homology-directed repair (HDR)^7^,^8^. NHEJ occurs throughout the cell cycle, whereas HDR occurs during the S- and G2 phases of the cell cycle^9^,^10^. NHEJ introduces small insertion or deletion (indel) mutations and disrupts targeted genes. In contrast, HDR utilizes homologous recombination-mediated strand exchange to repair DSBs^9^,^10^. CRISPR/Cas9-mediated gene knock-in utilizes the HDR machinery and enables the introduction of donor sequences into a specific genomic site with high efficiency^7^,^8^. This system has been shown to work in cultured cells, embryos, and organisms^7^,^8^,^11^,^12^,^13^, but not in postmitotic neurons.

There are several methods of introducing genes into neurons *in vivo*. Virus-mediated gene delivery systems are widely used, however, in such systems, the size of DNAs to be introduced is restricted^14^. *In utero* electroporation is another technique to introduce genes in a certain population of neurons in a cell-type-specific manner in the brain by transfecting genes into neural progenitor cells without limiting the size of DNA^15^,^16^,^17^.

Here, we demonstrate the HDR-mediated knock-in in brain neurons by the combined use of the CRISPR/Cas9 system and *in utero* electroporation technique. We show that the knock-in of the EGFP coding sequence in the site immediately after the first ATG codon of the β-actin gene enabled the visualization of the endogenous β-actin protein on the basis of EGFP fluorescence signals.

## Results

### Construction of CRISPR/Cas9 plasmids for gene knock-in at β-actin locus

**Figure 1:**
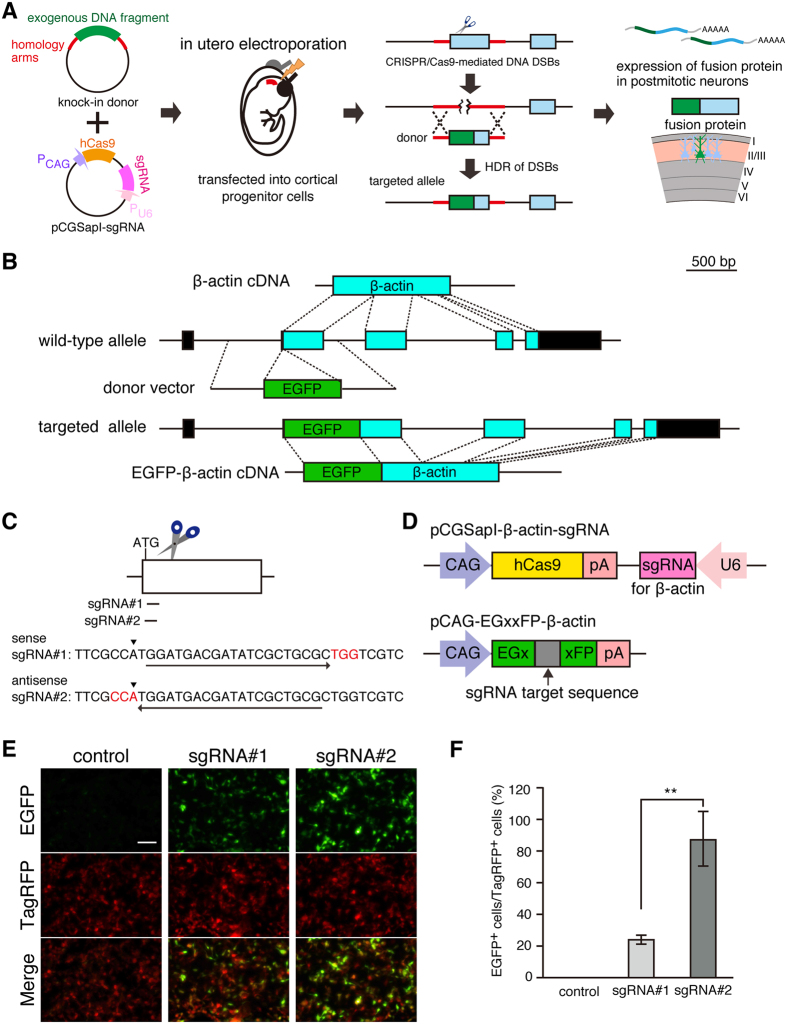
Construction and validation of plasmid vectors for CRISPR/Cas9-mediated knock-in. (**A**) Scheme for CRISPR/Cas9-mediated knock-in of exogenous DNA fragment in brain neurons. Donor vector contains exogenous DNA fragment flanked by homology arms. pCGSapI-sgRNA allows simultaneous expression of hCas9 and sgRNA. Donor and pCGSapI-sgRNA plasmid vectors are injected into lateral ventricles of embryos and transfected into cortical progenitor cells by *in utero* electroporation. CRISPR/Cas9-mediated HDR with donor vector results in knock-in of exogenous DNA fragment at a specific genomic locus and expression of fusion protein. (**B**) Schematic representation of β-actin cDNA, β-actin allele, donor vector, targeted allele, and EGFP-β-actin cDNA. Donor vector contains EGFP coding sequence flanked by 0.5-kb homology arms. (**C**) Genomic sequences and positions of two sgRNA target sites in exon 2 of β-actin gene. Arrows indicate target sequences and their direction. Arrowheads indicate the translation initiation codon of β-actin. PAM sequences are shown in red. (**D**) Schematic representation of pCGSapI-sgRNA and reporter plasmids. pCGSapI-β-actin-sgRNA plasmid contains expression cassettes of hCas9 and sgRNA against β-actin. The reporter plasmid pCAG-EGxxFP-β-actin contains sgRNA target sequence of β-actin. (**E**) Validation of sgRNA by HDR-mediated EGFP reconstitution. HEK293T cells were transfected with expression vectors for TagRFP, reporter, and hCas9/β-actin-sgRNA#1 or β-actin-sgRNA#2. Scale bar represents 100 μm. (**F**) Percentage of EGFP-positive cells relative to TagRFP positive cells (n = 6 each). All values represent mean ± SEM. **p < 0.01; Tukey’s test.

To perform gene knock-in in neurons in the brain, we combined two techniques: the use of the CRISPR/Cas9 system for genome editing and the *in utero* electroporation technique for transfection of plasmids into neurons in the brain (Fig. 1A). We chose the β-actin gene for CRISPR/Cas9-mediated gene knock-in (Fig. 1B). β-actin is highly expressed in neurons in the brain and plays an important role in cytoskeletal organization^18^. In this study, we attempted to perform EGFP knock-in at the β-actin locus by CRISPR/Cas9-mediated HDR with a donor plasmid vector (Fig. 1B).

To perform CRISPR/Cas9-mediated EGFP knock-in, we searched for the Cas9 target site of 20-nucleotide sequences adjacent to the PAM sequence in the vicinity of the first ATG codon in exon 2 of the β-actin gene. To minimize off-target effects, the CRISPR design tool (http://crispr.mit.edu/) was used^19^. We selected two candidate sgRNA sequences, namely sgRNA#1 and sgRNA#2 (Fig. 1C), and inserted them into pCGSapI, which allowed the coexpression of human codon-optimized Cas9 (hCas9) and sgRNA under the control of CAG (chicken β-actin promoter associated with the cytomegalovirus enhancer) and human U6 promoters, respectively (Fig. 1D).

### Validation of sgRNA against β-actin gene *in vitro*

To evaluate the on-target excision efficiency of sgRNAs, we applied an sgRNA validation system with which CRISPR/Cas9-mediated DSBs can be detected by observing green fluorescence signals reconstituted by HDR of an EGFP expression cassette^20^. To validate sgRNA against the β-actin gene, we constructed pCAG-EGxxFP-β-actin carrying the β-actin genomic fragment containing sgRNA target sites between 5′ and 3′ EGFP fragments with a 482-bp overlapping sequence (Fig. 1D). We cotransfected pCAG-TagRFP and pCAG-EGxxFP-β-actin together with pCGSapI-β-actin-sgRNA#1 or -sgRNA#2 into HEK293T cells. Forty-eight hours after transfection, reconstituted EGFP fluorescence signals were observed in the cotransfected HEK293T cells (Fig. 1E). In contrast, no EGFP fluorescence signals were detected in the HEK293T cells cotransfected with pCAG-TagRFP, pCAG-EGxxFP-β-actin and control pCGSapI lacking sgRNA. Next, we quantified the number of EGFP-positive HEK293T cells in transfection with each sgRNA candidate. The ratio of EGFP-positive to TagRFP-positive HEK293T cells was significantly higher for sgRNA#2 than for sgRNA#1 (87.3 ± 15.8% and 23.2 ± 2.55%, respectively; p < 0.01, Tukey’s test) (Fig. 1F), suggesting that sgRNA#2 induces DSBs more efficiently in this fragment. In the following experiments, we used sgRNA#2 for CRISPR/Cas9-mediated genome editing.

### CRISPR/Cas9-mediated EGFP knock-in at β-actin locus in cortical layer 2/3 pyramidal neurons

**Figure 2:**
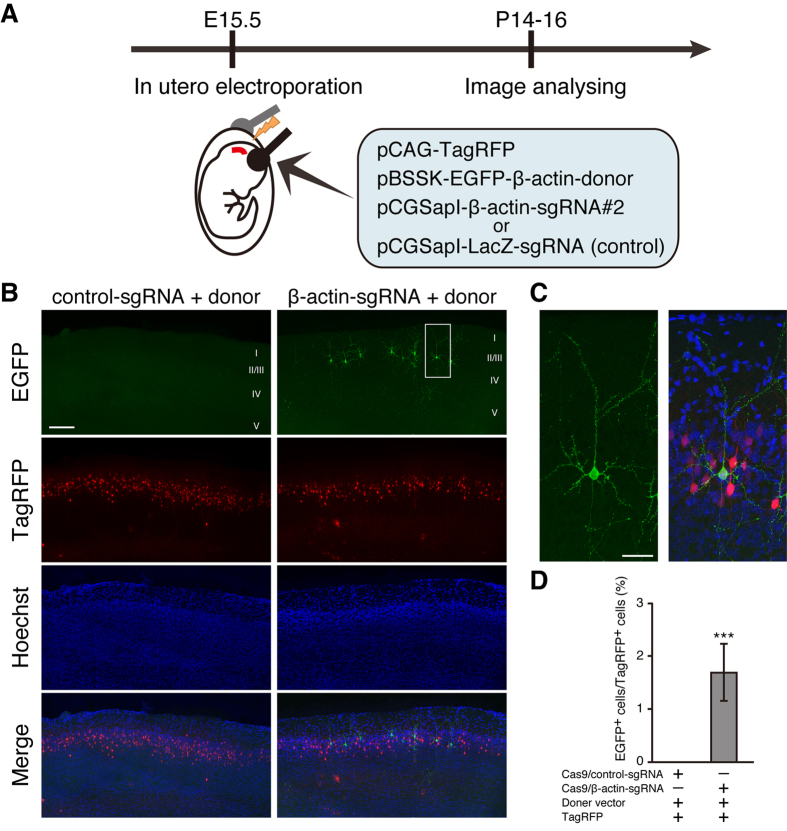
CRSIPR/Cas9-mediated knock-in of EGFP at β-actin locus in brain neurons. (**A**) Schematic representation of experimental procedure. Cortical progenitor cells are transfected with pCAG-TagRFP and pBSSK-EGFP-β-actin-donor together with pCGSapI-β-actin-sgRNA#2 or control pCGSapI-LacZ-sgRNA by *in utero* electroporation at E15.5. (**B**) Representative images of somatosensory cortex from transfected mice. Coronal brain sections were prepared at P14-16, followed by stained with Hoechst 33342. (**C**) High-resolution image of enclosed region in (**B**). (**D**) Percentage of EGFP-positive cells relative to TagRFP positive cells (n = 6 each). All values represent mean ± SEM. *** p < 0.001; Student’s t-test. Scare bars represent 200 μm in (**B**) and 50 μm in (**C**).

To examine whether CRISPR/Cas9-mediated HDR can be applied in neurons, we performed *in utero* electroporation to introduce pCAG-TagRFP and pBSSK-EGFP-β-actin-donor together with pCGSapI-β-actin-sgRNA#2 into neural progenitor cells in the ventricular zone at embryonic day 15.5 (E15.5) (Fig. 2A). LacZ-targeting sgRNA was used as a control^13^. These progenitor cells give rise to pyramidal neurons in layer 2/3 of the neocortex. TagRFP fluorescence signals were observed in layer 2/3 pyramidal neurons in the somatosensory cortex at postnatal day 14 (P14)-16 under both conditions. In contrast, EGFP fluorescence signals were detected in a sparse population of cortical layer 2/3 pyramidal neurons only in the mice transfected with pCGSapI-β-actin-sgRNA#2 (Fig. 2B). In addition, many punctate EGFP fluorescence signals were observed along dendrites, and a relatively strong and diffuse signal was observed in the soma (Fig. 2C). EGFP fluorescence signals were detected in approximately 1.7% of TagRFP-positive neurons only in the mice transfected with pCGSapI -β-actin-sgRNA#2 (Fig. 2D).

**Figure 3:**
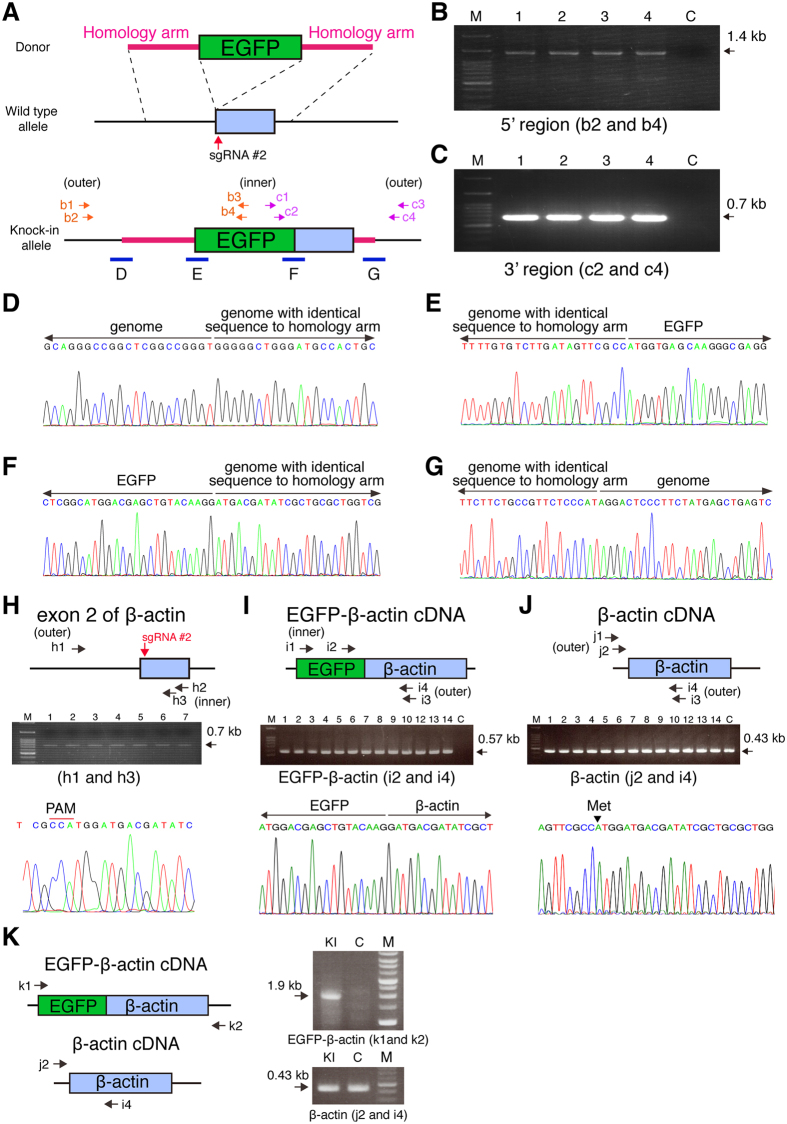
Validation of CRISPR/Cas9-mediated knock-in of EGFP at β-actin locus. (**A**) Schema represents EGFP donor, wild-type allele, and knock-in allele. Arrows indicate primers for nested genomic PCR. 5′ and 3′ targeting regions are amplified by nested PCR using primers b1-b4 and c1-c4, respectively. Blue bars indicate the boundaries between β-actin genome with identical sequence to homology arms and β-actin genome or EGFP coding sequence. (**B**) PCR amplification of 5′ targeting region using nested primers b2 and b4. (**C**) PCR amplification of 3′ targeting region using nested primers c2 and c4. (**D–G**) Representative sequence chromatograms of regions **D–G** in (**A**). (**H**) Validation of indel in EGFP-positive neurons. Semi-nested PCR was performed with indicated primers. Representative sequence chromatogram is shown. (**I**) Single-cell RT-PCR analysis of EGFP-β-actin mRNA in EGFP-positive neurons. The DNA fragments containing the junction between EGFP and β-actin were amplified with indicated nested primers. Representative sequence chromatogram is shown. (**J**) Single-cell RT-PCR analysis of β-actin mRNA in EGFP-positive neurons. The DNA fragments containing the sgRNA target site were amplified with indicated primers using the same samples in (**I**) as templates. Representative sequence chromatogram is shown. (**K**) Expression of full length EGFP-β-actin in mice transfected with donor, Cas9/β-actin-sgRNA, and TagRFP. The 1.9-kb DNA fragment containing the entire coding sequence of EGFP-β-actin was amplified with primers k1 and k2. For control, the β-actin fragment was amplified with primers j2 and i4. M, DNA size marker; C in (**B,C,I,J**), control EGFP-negative/TagRFP-positive cells; C in (**K**), control LacZ-sgRNA transfected brain region; KI, β-actin-sgRNA#2 transfected brain region.

To confirm the knock-in of EGFP in the β-actin gene in each neuron, we performed nested PCR using genomic DNA from single cells as templates. Genomic DNA samples isolated from individual EGFP-positive neurons using patch pipettes were subjected to nested PCR amplification with the inner primers in the EGFP sequence of the exogenous donor and the outer primers in the sequences flanking the homologous arms (Fig. 3A). The 1.4-kb and the 0.7-kb DNA fragments were amplified from all EGFP-positive neurons examined (n = 4 cells in each condition), but not from EGFP-negative/TagRFP-positive neurons (Fig. 3B,C). DNA sequencing analysis of the amplified DNA fragments revealed that the EGFP donor was correctly knocked-in at the β-actin gene locus in all EGFP-positive neurons (Fig. 3D–G and Supplementary Fig. 1). To examine whether EGFP knock-in occurs in both alleles or one allele, we performed nested PCR amplification of the β-actin gene locus containing the sgRNA targeting sequence. The 0.7-kb DNA fragments were amplified from all EGFP-positive neurons examined (n = 7 cells) (Fig. 3H), suggesting that EGFP knock-in did not occur in the other allele. The DNA sequence analysis of the amplified DNA fragments revealed that all these fragments have neither DNA fragment of plasmid donor vector nor indel mutations around the sgRNA target site (Fig. 3H). This result suggests that EGFP knock-in precisely occurs in one allele.

Next, we examined the expression of EGFP-β-actin mRNA in EGFP-positive neurons by single-cell RT-PCR analysis^21^. Cytoplasms of individual EGFP-positive neurons aspirated using patch pipettes were subjected to reverse transcription and nested PCR amplification with the inner primers in EGFP sequence and the outer primers in the β-actin cDNA sequence flanking the homologous arm (Fig. 3I). The 0.57-kb DNA fragments were amplified from all the EGFP-positive neurons examined, but not from EGFP-negative/TagRFP-positive neuron (Fig. 3I). The 0.57-kb DNA fragment was not amplified from pBSSK-EGFP-β-actin-donor (Supplementary Fig. 2). DNA sequencing analysis of the amplified DNA fragments revealed that EGFP was correctly fused to β-actin mRNA in frame in EGFP-positive neurons (Fig. 3I), suggesting that these neurons express EGFP-β-actin mRNA. Genomic DNA analysis suggests that the EGFP-positive neurons have both EGFP-knock-in and wild-type β-actin alleles (Fig. 3A–H). To examined the expression of β-actin mRNA in EGFP-positive neurons, we performed single-cell RT-PCR using β-actin cDNA-specific primers (Fig. 3J). The 0.43-kb DNA fragments containing the sgRNA targeting-sequence were amplified from both EGFP-positive and EGFP-negative/TagRFP-positive neurons (Fig. 3J). The amplified DNA fragments were analyzed by DNA sequencing. No indel mutations were detected in the amplified DNA fragments from EGFP-positive neurons (Fig. 3J). These results collectively suggest that almost all EGFP-positive neurons carry both the EGFP knock-in and wild-type alleles of the β-actin gene, and express both EGFP-β-actin and wild-type β-actin. To confirm the expression of full length EGFP-β-actin, we performed RT-PCR analysis using transfected brain region. The 1.9-kb fragment containing the entire coding region of EGFP-β-actin was amplified from the total RNA isolated from brain region transfected with pBSSK-EGFP-β-actin donor, pCGSapI-β-actin-sgRNA#2, and pCAG-TagRFP but not from that with control vector (Fig. 3K). Thus, CRISPR/Cas9-mediated EGFP knock-in occurred mostly in one allele of the β-actin gene in cortical layer 2/3 pyramidal neurons, and EGFP knock-in at the β-actin gene locus results in the expression of EGFP-β-actin. Collectively, knock-in efficiency was estimated to be approximately 1.7%. In the following experiments, we designated these EGFP-positive neurons as EGFP knock-in neurons.

**Figure 4:**
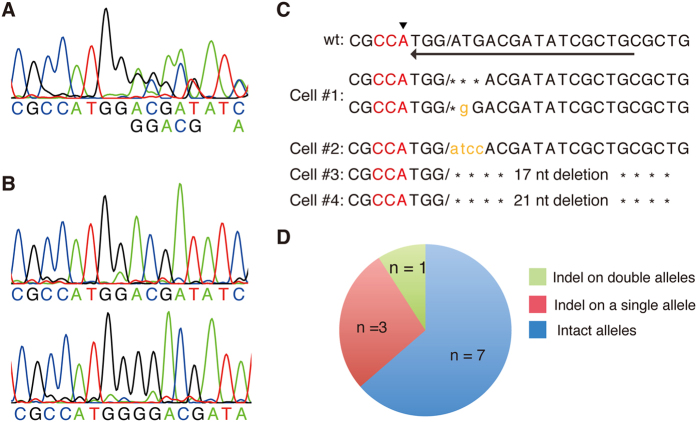
CRISPR/Cas9-mediated indel mutations in EGFP-negative/TagRFP-positive neurons. (**A**) The PCR amplified DNA fragments containing the β-actin-sgRNA target site from each single neuron were analyzed by DNA sequencing. Example of sequence chromatogram is shown. In this PCR product, moderate multiple peaks appeared from middle of the sequence. (**B**) PCR product in (**A**) was cloned into plasmid and sequenced. PCR product in (**A**) carried two different indel alleles. (**C**) Indel mutations in EGFP-negative/TagRFP positive neurons. Intact wild type sequence is shown on the top. Arrowhead and arrow indicate the first ATG codon of β-actin gene and the direction of sgRNA target sequence, respectively. Slashes represent predicted cleavage site. Cell#1 carried indel mutations in both alleles (see **A,B**). Cell#2-4 carried indel mutations in a single allele. (**D**) Frequency of indel mutations detected in the eleven EGFP-negative/TagRFP-positive neurons.

It has been reported that CRISPR/Cas9 introduces indel mutations at high frequencies. Thus, we examined indel mutations in individual EGFP-negative/TagRFP-positive neurons by single-cell PCR. Genomic DNA fragments containing the sgRNA targeting sequence were amplified by nested PCR and analyzed by DNA sequencing (Fig. 4A–C). Among 11 EGFP-negative/TagRFP-positive neurons, 3 cells carried the indel mutations at a single allele and 1 cell carried indel mutations at both alleles (Fig. 4D).

### Increase in EGFP knock-in efficiency by *in utero* electroporation of plasmid vectors at early developmental stage of brain

**Figure 5:**
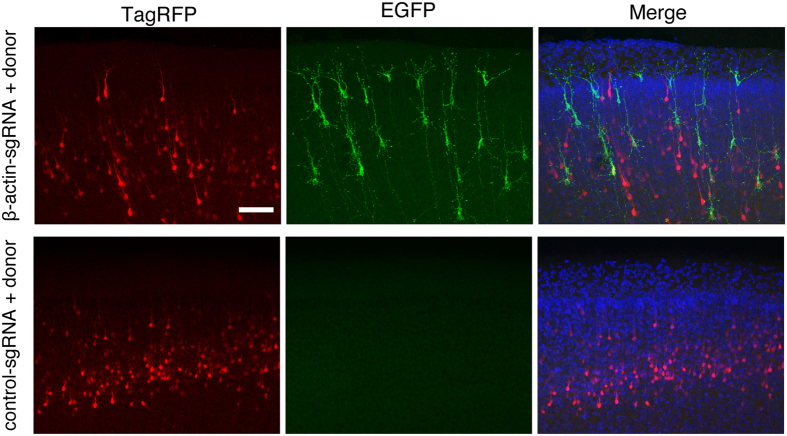
EGFP knock-in at the β-actin locus at an earlier developmental stage. Cortical progenitor cells were transfected with pCAG-TagRFP and pBSSK-EGFP-β-actin-donor together with pCGSapI-β-actin-sgRNA#2 or control pCGSapI-LacZ-sgRNA by *in utero* electroporation at E13.5. Representative images of somatosensory cortex from transfected mice. Coronal brain sections were prepared at P4-5, followed by stained with DAPI (blue). The images were merged on the right. Scare bar represents 100 μm.

Next, we performed *in utero* electroporation to introduce pCAG-TagRFP and pBSSK-EGFP-β-actin-donor together with pCGSapI-β-actin-sgRNA#2 or control pCGSapI-LacZ-sgRNA at E13.5. At P4-5, EGFP fluorescence signals were detected in a sparse population of cortical layer 2/3 neurons in mice transfected with pCGSapI-β-actin-sgRNA#2 (Fig. 5). EGFP fluorescence signals were also detected in deep cortical layers. No EGFP fluorescence signals were detected in the mice transfected with control pCGSapI-LacZ-sgRNA. In contrast to the result obtained with mice transfected at E15.5, most of all EGFP-positive cortical layer 2/3 pyramidal neurons did not express TagRFP (Fig. 5). Though there are many variations of the number of EGFP-positive cells in the cortical layer 2/3 in both conditions, maximal density of the EGFP-positive neurons was observed in mice transfected at E13.5 (0.9 cells/100 μm^2^). The EGFP-positive cells transfected at E13.5 were more widely and densely distributed in the cortex than those transfected at E15.5, suggesting that CRISPR/Cas9-mediated EGFP knock-in is more efficient at a early developmental stage of the brain at which more neural progenitor cells are still dividing.

### EGFP knock-in exerts little effect on spine morphology of cortical layer 2/3 pyramidal neurons

**Figure 6:**
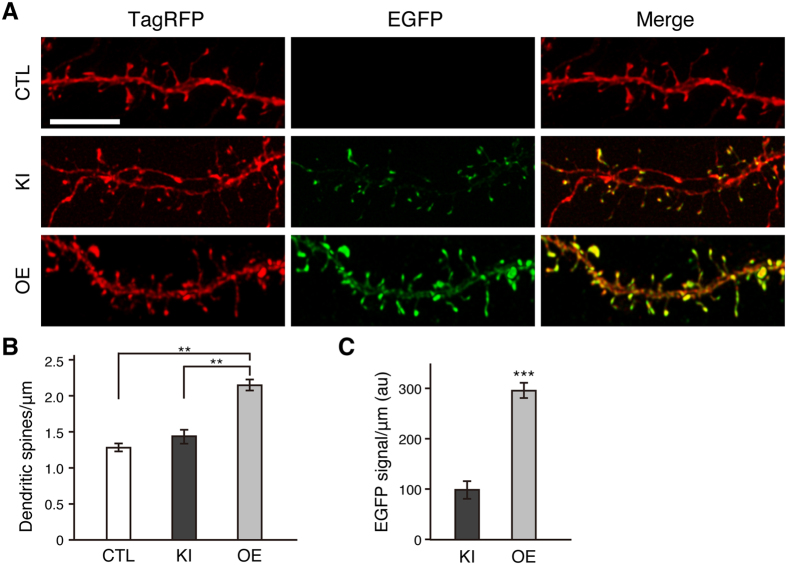
Effects of EGFP-tagged endogenous β-actin and exogenously overexpressed EGFP-β-actin on spine morphology of cortical layer 2/3 pyramidal neurons. (**A**) Representative images of dendritic spines of control, EGFP knock-in and exogenous EGFP-β-actin overexpressed neurons. Scale bar represents 5 μm. (**B**) Spine densities of basal dendrites of control, EGFP knock-in, and exogenous EGFP-β-actin overexpressed neurons (n = 20 from 3 mice, respectively). (**C**) Intensities of EGFP signals on dendrites of EGFP knock-in and exogenous EGFP-β-actin overexpressed neurons (n = 10 from 3 mice, respectively). CTL, control; KI, EGFP knock-in; OE, exogenous EGFP-β-actin overexpression. All values represent mean ± SEM. ***p < 0.001 and **p < 0.01, respectively; Tukey’s test (**B**) or Student’s t-test (**C**).

EGFP knock-in at the β-actin locus resulted in the expression of EGFP-β-actin (Fig. 2). In EGFP knock-in neurons, strong EGFP fluorescence signals densely accumulated in the dendritic spine heads (Fig. 6A). The similar localization was observed in neurons transfected with HA-tag donor vector in which the coding sequence of EGFP in pBSSK-EGFP-β-actin donor was replaced by HA-tag coding sequence (Supplementary Fig. 3), suggesting that EGFP tagging on the β-actin protein has little effect on its localization. Next, we examined the effects of EGFP knock-in on the dendritic spine morphology of cortical layer 2/3 pyramidal neurons. In EGFP knock-in neurons, strong EGFP fluorescence signals densely accumulated in the dendritic spine heads (Fig. 6A). We then analyzed the spine density in EGFP-expressing neurons at P14-16, which is the period when the spine density of basal dendrites of cortical layer 2/3 pyramidal neurons reaches a plateau^22^. The spine densities of basal dendrites of cortical layer 2/3 pyramidal neurons were comparable between control and EGFP knock-in neurons (Fig. 6A,B). This result suggests that EGFP knock-in in the β-actin gene exerts little effect on spine formation. To examine whether the overexpression of EGFP-β-actin affects the dendritic spine morphology, neuronal progenitor cells were transfected with an expression vector for EGFP-β-actin. In exogenous-EGFP-β-actin-overexpressing neurons, the intensity of EGFP fluorescence signals along the dendrites increased by three fold compared with that in EGFP knock-in neurons (p < 0.001) (Fig. 6A,C). The spine density significantly increased in exogenous-EGFP-β-actin-overexpressing neurons (p < 0.01) (Fig. 6A,B).

### EGFP knock-in exerts little effect on neuronal functions in cortical layer 2/3 pyramidal neurons

**Figure 7:**
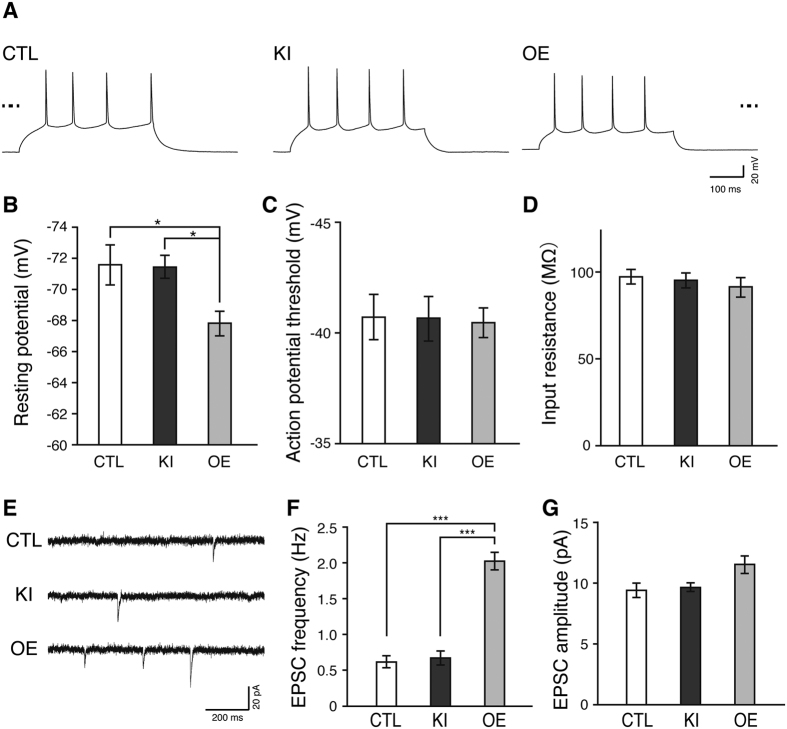
Effect of EGFP knock-in and exogenous EGFP-β-actin overexpression on physiological properties of cortical layer 2/3 pyramidal neurons. (**A**) Representative traces of firing pattern of control, EGFP knock-in, and exogenous EGFP-β-actin overexpressed neurons. Traces of membrane potentials of all the neurons were placed based on the actual value of membrane potential. Bold dashed lines on the sides represent 0 mV. (**B–D**) Basic electrophysiological properties of control, EGFP knock-in, and exogenous EGFP-β-actin overexpressed neurons. Resting membrane potential, action potential threshold, and input resistance of control (n = 13 neurons from 4 mice), EGFP knock-in (n = 15 from 4 mice), and exogenous EGFP-β-actin overexpressed neurons (n = 15 from 4 mice) are shown in (**B–D**), respectively. (**E**) Representative traces of EPSCs from control, EGFP knock-in, and exogenous EGFP-β-actin overexpressed neurons. (**F,G**) EPSCs in control, EGFP knock-in, and exogenous EGFP-β-actin overexpressed neurons. EPSCs frequency and amplitude of control (n = 9 neurons from 3 mice), EGFP knock-in (n = 11 from 3 mice), and exogenous EGFP-β-actin overexpressed neurons (n = 10 from 3 mice) are shown in (**F,G**), respectively. CTL, control; KI, EGFP knock-in; OE, EGFP-β-actin overexpression. All values represent mean ± SEM. ***p < 0.001 and *p < 0.05; Tukey’s test.

To examine whether EGFP knock-in affects the functions of neurons, we measured the electrophysiological properties of neurons in acute brain slices from P14-16 mice by patch clamp recording (Fig. 7). The resting membrane potential, action potential threshold, and input resistance were comparable between EGFP knock-in and control neurons (Fig. 7A–D). In contrast, the resting membrane potential was altered in EGFP-β-actin-overexpressing neurons (p < 0.05), whereas the action potential threshold and input resistance remained unchanged (Fig. 7A–D). We next examined excitatory postsynaptic currents (EPSCs) in EGFP knock-in and EGFP-β-actin-overexpressing neurons. The frequency and amplitude of EPSCs were comparable between EGFP knock-in and control neurons (Fig. 7E–G). In contrast, EGFP-β-actin-overexpressing neurons showed an increase in the frequency (p < 0.01), but not in the amplitude, of EPSCs (Fig. 7E–G). These results suggest that EGFP knock-in in the β-actin gene exerts no detectable effects on neuronal and synaptic functions.

## Discussion

CRISPR/Cas9-mediated gene knock-in is a powerful technique for precise gene modification in somatic cells, which is time- and cost-saving compared with conventional procedures, such as gene targeting in ES cells. Here, we demonstrated CRISPR/Cas9-mediated EGFP knock-in in cortical layer 2/3 pyramidal neurons by the combined use of the CRISPR/Cas9 system and *in utero* electroporation technique, resulting in the expression of the EGFP-tagged endogenous β-actin protein.

CRISPR/Cas9-mediated gene knock-out by error-prone NHEJ has been achieved in a wide variety of cells in different species^5^,^6^. NHEJ functions in both dividing and nondividing cells, whereas HDR functions only in dividing cells^9^,^10^. CRISPR/Cas9-mediated gene knock-out in postmitotic neurons *in vivo* has been reported such as mouse cortical neurons, mouse hippocampal dentate gyrus granule neurons, and mouse cerebellar Purkinje cells^13^,^23^,^24^,^25^,^26^,^27^,^28^. CRSIPR/Cas9-mediated gene knock-in has been shown in dividing cells, but not in postmitotic neurons. In the present study, we demonstrated the genome-edited single postmitotic neurons by introducing the CRISPR/Cas9 and donor plasmids into the cortical dividing progenitor cells using *in utero* electroporation technique. Very recently endogenous protein tagging of several proteins in various brain regions by CRSIPR/Cas9-mediated HDR with synthetic single-stranded oligodeoxynucleotide and plasmid-based donor has also been reported, however, sequence information of edited genomic region of knock-in neurons at single cell level was not presented^29^. Although we demonstrated one example of protein tagging of β-actin in cortical layer 2/3 pyramidal neurons using plasmid donor vector, our study further investigated the sequence of edited genomic region and transcripts with precision at single cell level as well as neuronal properties.

We estimated the efficiency of EGFP knock-in to be approximately 1.7%. Interestingly, all EGFP-positive neurons examined had both EGFP knock-in and wild-type alleles of the β-actin gene (Fig. 3). There seems to be two possibilities. One is that the homozygous modified of the β-actin locus is toxic to neurons. But this is unlikely. No abnormalities in cortical structures and neurons were detected in the CNS specific β-actin knock-out mice^30^. Another is that the ratio of CRISPR/Cas9-induced DNA cleavage in a single β-actin allele is higher than that in both alleles. Under our experimental conditions, approximately 27% (3 out of 11 cells) of EGFP-negative/TagRFP-positive neurons had heterozygous indel mutations and 9% (1 out of 11 cells) of EGFP-negative/TagRFP-positive neurons had homozygous indel mutations (Fig. 4). Thus, we consider the latter interpretation to be more reasonable.

EGFP-tagged endogenous β-actin localized at the head of dendritic spines (Fig. 6), consistent with the localization of immunolabeled β-actin^31^,^32^,^33^. Several evidences suggest that CRISPR/Cas9 systems have off-target cleavage activity at similar gene sequences, and off-target effect remains a major concern^34^,^35^,^36^. β-actin sgRNA used in our study had little effect on morphological and electrophysiological properties of cortical layer 2/3 pyramidal neurons, suggesting there are little or no off-target effects, at least, of this sgRNA on neuronal functions. Also, EGFP-tagging onto endogenous β-actin exerted little effect on cytoskeletal organization. Exogenously expressed EGFP-β-actin has been shown to assemble into filaments^18^,^37^.

Overexpression of exogenous proteins often perturbs normal cellular functions. Indeed in this study, exogenously overexpressed EGFP-β-actin in neurons altered the spine density, and neuronal and synaptic functions (Figs 6 and 7). Increased spine density was also observed in cultured rat hippocampal neurons overexpressing EGFP-β-actin^38^. On the other hand, hCas9 presumably overexpressed in EGFP knock-in cortical layer 2/3 pyramidal neurons exerted no adverse effect on neuronal physiology (Fig. 7). Consistent with our observation, constitutive Cas9 expression itself does not affect electrophysiological properties in mouse CA1 pyramidal neurons^13^. Thus, the CRISPR/Cas9-mediated knock-in technique is highly advantageous for the direct visualization of an endogenous protein by fluorescent protein tagging or small-epitope tagging with an appropriate expression level without altering neuronal functions.

Our simple and rapid technique provides a potential tool to elucidate the localization of various endogenous proteins in neurons without using antibodies. This technique can also be used for introducing a specific mutation into genes of interest to investigate molecular functions in brain neurons. Our results of sequence of edited genomic region and transcripts as well as physiological properties in EGFP knock-in neurons at single cell level would provide useful information for application of this method in brain neurons.

## Methods

### Construction of expression vectors

The entire coding sequence of TagRFP was amplified by PCR using pTagRFP-C (Evrogen) as a template and cloned into pCAG-1^39^ to yield pCAG-TagRFP. The 600-bp genomic DNA fragment containing the translation initiation codon of the β-actin was amplified by PCR with primers 5′-CTGCAGGGTCCGCCTCCGGGCCAGCG-3′ and 5′-GAATTCGGTGAGCAGCACAGGGTGCTC-3′ using ICR mouse genomic DNA as a template and cloned into PstI-EcoRI sites of pCAG-EGxxFP (#50716, Addgene) to yield pCAG-EGxxFP-β-actin. The 467-bp DNA fragment carrying human U6 promoter and sgRNA scaffold was amplified by PCR using pgRNA_GFP-T1 (#41819; Addgene) as a template, and cloned into pBluescript II SK(+) (Stratagene) to yield pGSapI. The 6.5-kb NotI fragment carrying CAG-NFCas9pA^40^ was cloned into NotI site of pGSapI, followed replaced the gRNA fragment of pGSapI with annealed oligonucleotides to yield pCGSapI. The sequences of oligonucleotides were as follows: 5′-GAAGAGCCTCGAGGAATTCGCTCTTC-3′ and 5′-GAAGAGCGAATTCCTCGAGGCTCTTC-3′. Annealed oligonucleotides were inserted into SapI site of pCGSapI to yield pCGSapI-β-actin-sgRNA#1, pCGSapI-β-actin-sgRNA#2, and pCGSapI-LacZ-gRNA, respectively. The sequences of oligonucleotides were as follows: β-actin-sgRNA#1, 5′-ACCGGGATGACGATATCGCTGCGCG-3′ and 5′-AAACGCGCAGCGATATCGTCATCCC-3′; β-actin-sgRNA#2, 5′-ACCGCGCAGCGATATCGTCATCCAG-3′ and 5′-AAACTGGATGACGATATCGCTGCGC-3′; LacZ-sgRNA, 5′-ACCGTGCGAATACGCCCACGCGATG-3′ and 5′-AAACATCGCGTGGGCGTATTCGCAC-3′. The 1.0-kb genomic DNA fragment carrying exon2 of the β-actin gene was amplified by PCR with primers 5′-GACTCACTATAGGGCTGGGATGCCACTGCGCGTGC-3′ and 5′-ACCCTCACTAAAGGGTGGGAGAACGGCAGAAGAAA-3′ using ICR mouse genomic DNA as template, and cloned into multiple cloning site of pBluescript SK(+) (Stratagene) using GeneArt Seamless Cloning and Assembly Kit (Invitrogen) to yield pBSSK-β-actin. The coding sequence of enhanced green fluorescent protein (EGFP) was amplified by PCR using pEGFP-C1 (Clontech) as template and inserted in-frame into the translation initiation codon of the β-actin of pBSSK-β-actin to yield pBSSK-EGFP-β-actin-donor. pBSSK-EGFP-β-actin-donor contains EGFP cDNA, the 508-bp upstream and the 530-bp downstream genomic sequences from the translation initiation codon of the β-actin. The coding sequence of EGFP in pBSSK-EGFP-β-actin-donor was replaced by the DNA fragment encoding 2xHA tag (YPYDVPDYAYPYDVPDYA) to yield pBSSK-HA-tag-β-actin-donor. The entire coding sequence of the β-actin was amplified by RT-PCR using total RNA isolated from ICR mouse brain as template. The β-actin cDNA was fused in frame with the EGFP coding sequence by PCR and subcloned into EcoRI-XhoI sites of pCAG-1 to yield pCAG-EGFP-β-actin.

### Cell culture

HEK293T cells were transfected with pCAG-EGxxFP-β-actin and pTagRFP-C together with pCGSapI-β-actin-sgRNA#1 or - sgRNA#2. Forty-eight hours after transfection, cells were fixed with 4% paraformaldehyde (PFA) and stained with Hoechst 33342 (WAKO). Fluorescence images were taken with the Operetta high throughput imaging system (Perkin Elmer). To quantify ratio of EGFP positive cells to TagRFP positive cells, the images were analyzed by Operetta imaging software 2.6 (Perkin Elmer).

### *In utero* electroporation

*In utero* electroporation was performed as essentially described previously^15^. Briefly, pregnant ICR mice at E15.5 or E13.5 were anesthetized, and the uterine horns were exposed. Approximately 1 μl of DNA solutions containing 0.01% fast green were injected into the lateral ventricles of embryos using a pulled borosilicate glass capillaries (B120F-4; World Precision Instruments). The DNA solutions contained 1.5 μg/ul Cas9/sgRNA plasmid, 1.5 μg/ul pBSSK-EGFP-β-actin-donor, and 1.0 μg/ul pCAG-TagRFP or 1.0 μg/ul pCAG-EGFP-β-actin and 1.0 μg/ul pCAG-TagRFP. The head of embryo in the uterus was placed between tweezers-type electrodes with 5 mm diameter (CUY650P5; NEPA Gene). Each injected embryos was subjected to five square electric pulses (35 V, 50 msec, 1 Hz) using electroporator (CUY21E; NEPA Gene). After electroporation, the embryos were returned to the abdominal cavity to allow continuous development. Transfected pups were identified at P0-2 by TagRFP signals through scalp using a fluorescence stereomicroscope. The animal protocol was approved by the Animal Care and the Use Committee of Shinshu University. The methods were carried out in accordance with the Regulations for Animal Experimentation of Shinshu University.

### Histological analysis

Under deep anesthesia, ICR mice were fixed transcardially with 4% PFA in phosphate-buffered saline, pH 7.4. Fifty- or 100-μm-thick coronal sections were prepared with microslicer (VT1200S; Leica Biosystems). Sections were stained with Hoechst 33342 and mounted. Fluorescence images of 100-μm-thick sections were taken with a confocal laser-scanning microscope (TCS SP8; Leica Microsystems) using HC PL APO CS2 20×/0.75 NA multiple immersion lens (Leica Microsystems). Images were taken using the tile scan function of the confocal microscope. Fluorescence images of 50-μm-thick sections were taken using HC PL APO CS2 100×/1.40 NA oil immersion lens (Leica Microsystems). Basal dendrites of the cortical layer 2/3 pyramidal neurons were randomly sampled and captured. Image stacks were deconvolved using Huygens Essential version (Scientific Volume Imaging). Spines on tertiary dendrites were identified and counted in the 3D projection images with respect to each mouse. For immunohistochemical analysis, 50-μm-thick sections were prepared as described above, and immunostained with rabbit anti-HA antibody (Covance), followed by incubation with Alexa Fluor 488-conjugated anti-rabbit antibody (Invitrogen) and DAPI. Images were taken as described above.

### Single-cell genomic PCR

Nucleus of EGFP-positive or EGFP-negative/RFP-positive neurons were harvested with patch pipettes and put into 0.2-ml PCR tubes containing PBS. Proteinase K (1 mg/ml, Toyobo) was added to the tubes, incubated at 55 °C for 15 min, by followed incubation at 75 °C for 20 min. The 5′ boundary region between the donor and β-actin genome was amplified by nested PCR. The first PCR was performed with primers 5′-ACAGCTTCTTTGCAGCTCCT-3′ (b1) and 5′-TTCTTCTGCTTGTCGGCCAT-3′ (b3). The second nested PCR was performed with primers 5′-CTTCGCTCTCTCGTGGCTAG-3′ (b2) and 5′-TTCTGCTTGTCGGCCATGAT-3′ (b4). The 3′ boundary region between the donor and β-actin genome was amplified by nested PCR. The first PCR was performed with primers 5′-GAGCAAAGACCCCAACGAGA-3′ (c1) and 5′-CAGTGTGCTGGGAGTCTCAG-3′ (c3). The second nested PCR was performed with primers 5′-CACATGGTCCTGCTGGAGTT-3′ (c2) and 5′-CCTCGTCTGGGAAAGAGCAG-3′ (c4). The genomic DNA fragment carrying the sgRNA target site was amplified by semi-nested PCR. The first PCR was performed with primers 5′-CACATGGTCCTGCTGGAGTT-3′ (h1) and 5′-CACATGGTCCTGCTGGAGTT-3′ (h2). The second semi-nested PCR was performed with primers h1 and 5′-CGATGGAGGGGAATACAGCC-3′ (h3). PCR was performed under the following conditions: the first PCR, one cycle of 98 °C for 2 min; 40 cycles of 94 °C for 10 sec, 63 °C for 20 sec and 68 °C for 30 sec; the second PCR, one cycle of 98 °C for 2 min; 30 cycles of 94 °C for 10 sec, 63 °C for 20 sec and 68 °C for 30 sec. PCR products were purified using a FastGene Gel/PCR Extraction Kit (Nippon Genetics) and analyzed by DNS sequencing. PCR products were subcloned into plasmid using Zero Blunt TOPO cloning kit (ThermoFisher Scientific) and analyzed by DNA sequencing. All PCR was performed using KOD FX Neo polymerase (TOYOBO).

### RT-PCR

Cytoplasms of EGFP-positive or EGFP-negative/RFP-positive neurons were aspirated through patch pipettes and the aspirated cytoplasms of individual neurons were directly put into a 0.2-ml PCR tube containing 2× RT master mix (High Capacity cDNA Reverse Transcription Kits; Applied Biosystems). cDNA was synthesized according to the manufacturer′s instruction. β-actin and EGFP-β-actin fragments were amplified by nested PCR. The first PCR was performed with primers 5′-TTTGCAGCTCCTTCGTTGCCGGTC-3′ (j1) and 5′-CCTGGATGGCTACGTACATGG-3′ (i3), 5′-ACGTAAACGGCCACAAGTTC-3′ (i1) and i3, respectively. The second PCR was performed with primers 5′-TTCGTTGCCGGTCCACACCC-3′ (j2) and 5′-ACATGGCTGGGGTGTTGAAG-3′ (i4), 5′-ACCACTACCAGCAGAACACC-3′ (i2) and i4, respectively, using diluted the initial reactions as templates. PCR was performed under the following conditions: the first PCR, one cycle of 98 °C for 2 min; 40 cycles of 94 °C for 10 sec, 60 °C for 30 sec and 68 °C for 30 sec; the second nested PCR, one cycle of 98 °C for 2 min; 30 cycles of 94 °C for 10 sec, 60 °C for 30 sec and 68 °C for 30 sec. PCR products were purified using a FastGene Gel/PCR Extraction Kit and analyzed by DNA sequencing. Total RNA was isolated from brain region transfected with pCAG-TagRFP and pBSSK-EGFP-β-actin-donor together with pCGSapI-β-actin-sgRNA#1 or control pCGSapI-β-LacZ-sgRNA using TRIZOL reagent (Thermo Fisher Scientific) according to the manufacturer’s instruction. cDNA was synthesized using Superscript III First-Strand Synthesis Super Mix (Invitrogen) according to the manufacturer’s instruction. The entire coding region of EGFP-β-actin was amplified by PCR with primers 5′-ATGGTGAGCAAGGGCGAGGAG-3′ and 5′-GCGCAAGTTAGGTTTTGTCAAAG-3′ using the following condition: one cycle of 94 °C for 2 min; 35 cycles of 98 °C for 10 sec, 65 °C for 30 sec, and 68 °C for 1 min. All PCR was performed using KOD FX Neo polymerase.

### Electrophysiology

P14-P16 mouse brains were removed and placed immediately in ice-cold slicing artificial corticospinal fluid (ACSF, in mM: 85 NaCl, 75 sucrose, 2.5 KCl, 1.25 NaH_2_PO_4_, 24 NaHCO_3_, 25 glucose, 0.5 CaCl_2_, and 4 MgCl_2_) saturated with 95% O_2_/5% CO_2_ for 2 min. The chilled brains were trimmed coronally with razor blades and placed in a vibratome chamber (Campden 7000smz). Three hundred fifty-μm-thick coronal sections were transferred to a recovery chamber filled with recording ACSF (in mM: 126 NaCl, 2.5 KCl, 1.25 NaH_2_PO_4_, 26 NaHCO_3_, 10 glucose, 2 CaCl_2_, and 2 MgCl_2_), followed by incubated at 32 °C for 30 min, and then at room temperature for 1 hour. Pyramidal neurons were patched with glass pipettes (4-8 Mohm) filled with intra-cellular solution (in mM: 130 K-gluconate, 6 KCl, 10 HEPES, 1 EGTA, 2.5 MgCl_2_, 2 magnesium ATP, 0.5 sodium GTP, 10 phosphocreatine sodium, 290 mOsm) under a fluorescence microscopy (BX50-WI, Olympus). Resting membrane potential was measured immediately after establishing whole cell recording in a current clamp mode. Action potentials were induced by injecting 500 ms duration of positive currents. Membrane potential at which the temporal rate of the potential reached to 10 mV/ms^41^ was defined as action potential threshold. Spontaneous EPSCs was recorded in a voltage clamp mode with holding potential at 60 mV, the reversal potential of GABA_A_ receptor. Input resistance was calculated with the current responses to the 2 mV step pulse. All data were acquired with EPC10 double amplifier (HEKA) operated by Patch Master software (HEKA). Access resistance was monitored throughout the recording, and cells with access resistance over 25 MΩ or changed over 25% were rejected. Data analysis was performed with Mini Analysis Program (Synaptosoft) and Igor Pro (WaveMetrics).

### Statistical analysis

Statistical significance was evaluated by one-way ANOVA followed by *post hoc* Tukey’s test for multiple comparisons of mean values. Statistical significance in mean values of two-sample was evaluated by Student’s *t* test. Statistical significance was assumed when p < 0.05. All data are shown as means ± SEM to indicate the precision of estimated mean of population.

## Additional Information

**How to cite this article**: Uemura, T. *et al*. Fluorescent protein tagging of endogenous protein in brain neurons using CRISPR/Cas9-mediated knock-in and *in utero* electroporation techniques. *Sci. Rep.* **6**, 35861; doi: 10.1038/srep35861 (2016).

**Publisher’s note:** Springer Nature remains neutral with regard to jurisdictional claims in published maps and institutional affiliations.

## Supplementary Material

Supplementary Information
