# Supplementary material for: Fluorescent protein tagging of endogenous protein in brain neurons using CRISPR/Cas9-mediated knock-in and in utero electroporation techniques
Source: Sci Rep. 2016 Oct 26;6:35861. doi: 10.1038/srep35861 (PMC5080626; doi:10.1038/srep35861)
Supplement: Supplementary Information [file srep35861-s1.pdf]

Fluorescent protein tagging of endogenous protein in brain neurons using CRISPR/Cas9-mediated knock-in and *in utero* electroporation techniques

Takeshi Uemura[1,2,3,7,*], Takuma Mori[1,7], Taiga Kurihara[1], Shiori Kawase[1,3], Rie Koike[1,3], Michiru Satoga[1], Xueshan Cao[1], Xue Li[1], Toru Yanagawa[4], Takayuki Sakurai[5], Takayuki Shindo[5] & Katsuhiko Tabuchi[1,2,6,*]

[1]Department of Molecular and Cellular Physiology, Institute of Medicine, Academic Assembly, Shinshu University, Nagano 390-8621, Japan
[2]Institute for Biomedical Sciences, Interdisciplinary Cluster for Cutting Edge Research, Shinshu University, Nagano 390-8621, Japan
[3]CREST, JST, Saitama 332-0012, Japan
[4]Department of Oral and Maxillofacial Surgery, Faculty of Medicine, University of Tsukuba, Ibaraki 305-8575, Japan
[5]Department of Cardiovascular Research, Shinshu University Graduate School of Medicine, Nagano 390-8621, Japan
[6]PRESTO, JST, Saitama 332-0012, Japan
[7]These authors contributed equally to this work.

[*]Correspondence and requests for materials should be addressed to T.U. (email: tuemura@shinshu-u.ac.jp) or to K.T. (email: ktabuchi@shinshu-u.ac.jp).

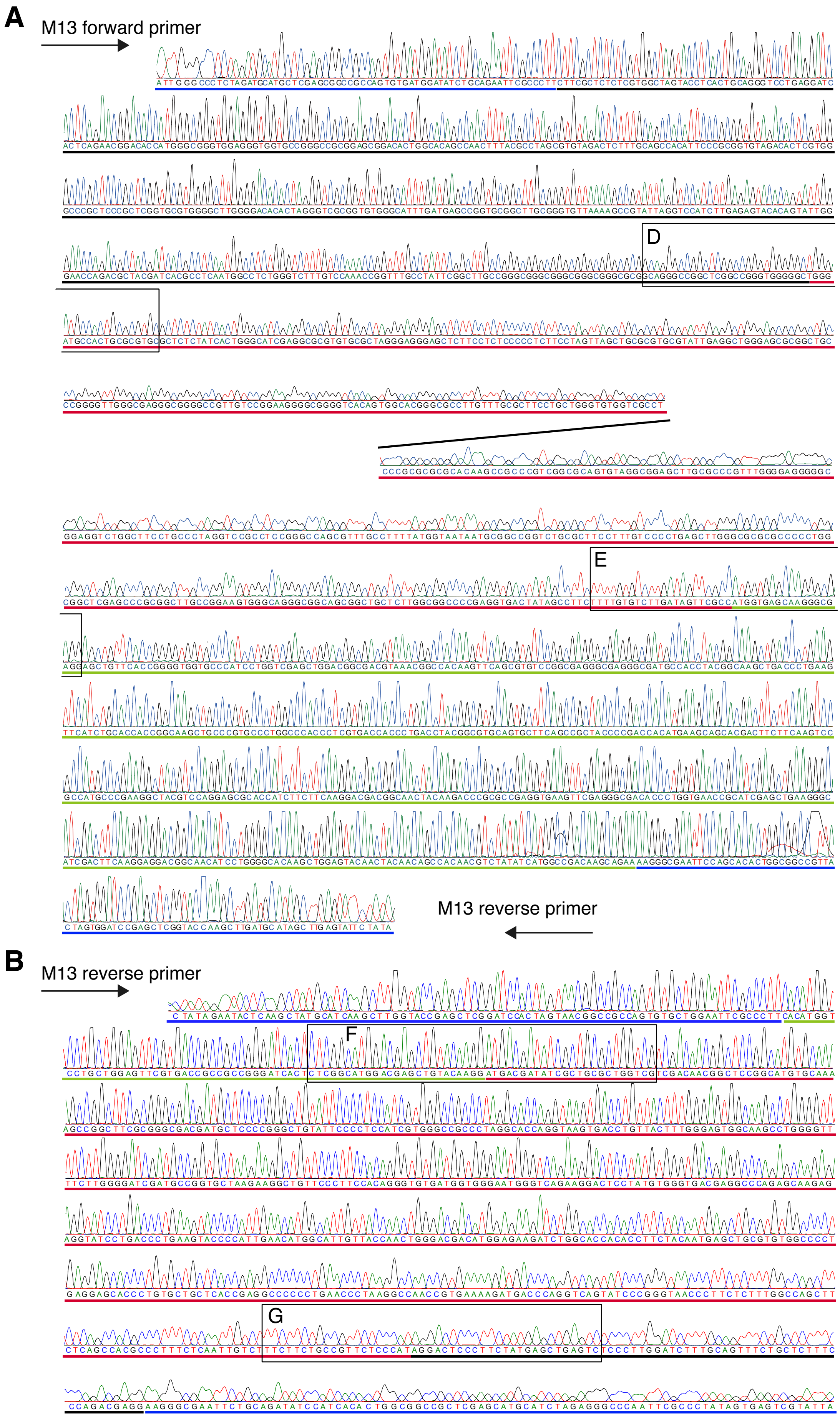

Supplementary Figure 1. Representative full DNA sequences and their chromatograms of the nested PCR-amplified DNA fragments, related to Figure 3A-G. (A) The PCR-amplified DNA fragments using nested primers b2 and b4 were subcloned into cloning vector and subjected to DNA sequencing using M13 forward and reverse sequencing primers. These two sequences are partially overlapped at both ends and collectively converted the entire region. (B) The PCR-amplified DNA fragments using nested primers c2 and c4 were subcloned into cloning vector and subjected to DNA sequencing using M13 reverse sequencing primer. Blue, black, red, and green underlines indicate cloning vector, genome, genome with identical sequence to homology arm, and EGFP coding sequences, respectively. Box regions indicate the representative sequence D-G in Figure 3D-G.

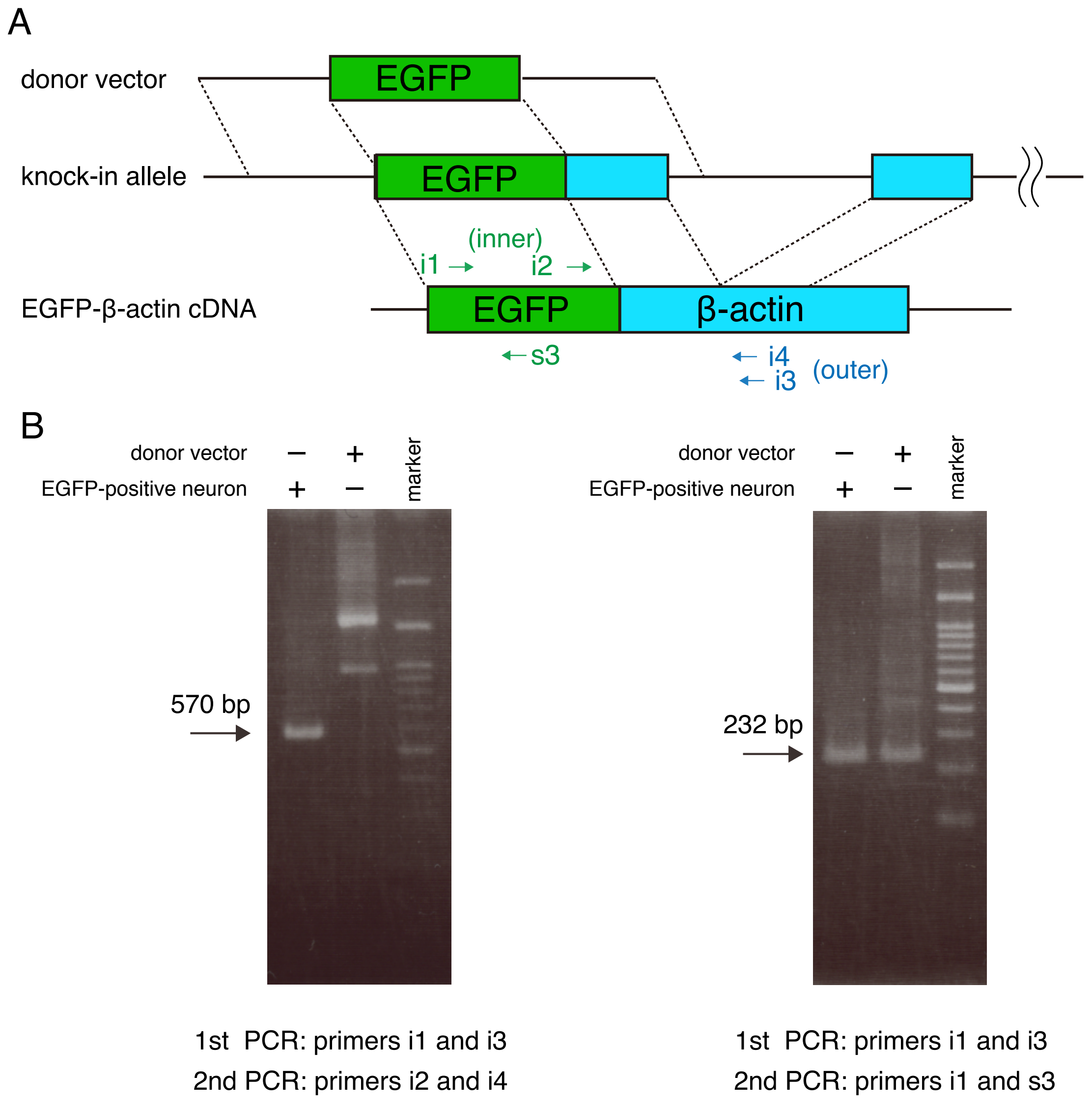

Supplementary Figure 2. Control experiments, related to Figure 3I. (A) Schematic representation of donor vector, EGFP knock-in allele, and EGFP-β-actin cDNA. Arrows indicate primers for nested RT-PCR. (B) Nested RT-PCR for detecting EGFP-β actin mRNA. The 0.57-kb fragment was amplified from EGFP-positive neuron but not from donor vector by using inner and outer primers (i2 and i4) (left panel). On the other hand, the 0.23-kb fragment were amplified from both EGFP-positive neuron and donor vector by using inner primer set (i1 and s3) (right panel) .

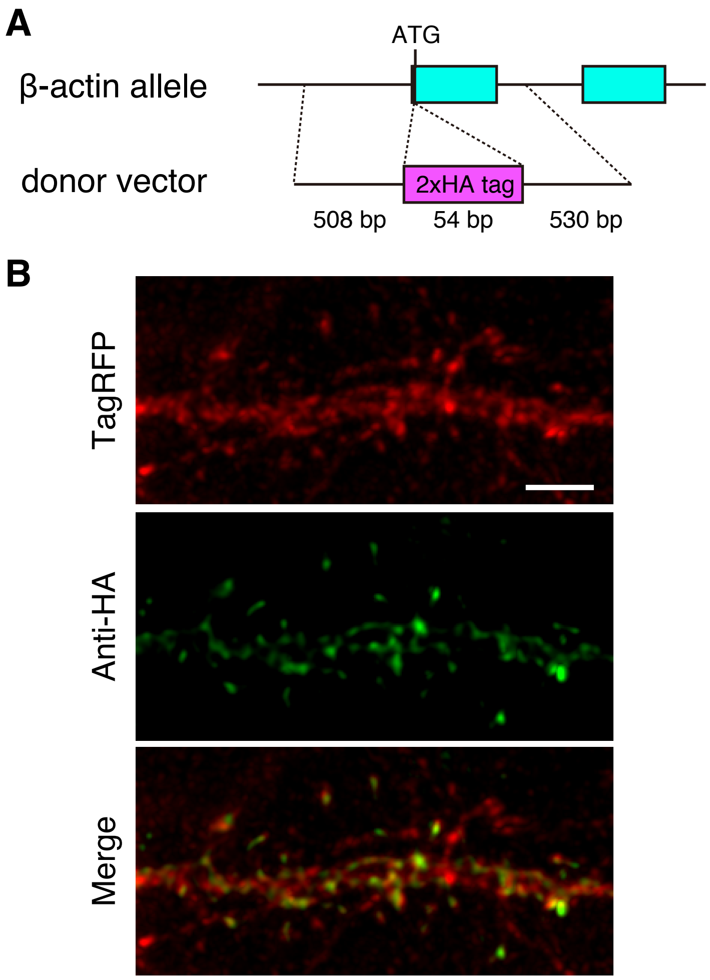

Supplementary Figure 3. Localiztion of HA-tagged endogenous β-actin protein, related to Figure 6. (A) Schematic representation of β-actin allele and donor vector for 2xHA-tag knock-in. (B) Representative images of dendrite of cortical layer 2/3 neuron. Cortical progenitor cells were transfected with pCAG-TagRFP and pBSSK-HA-tag-β-actin donor together with pCGSapI-β-actin-sgRNA#2 by in utero electroporation at E15.5. Coronal brain section was stained with anti-HA-tag antibody. Scare bar represents 2 μm.
